# Improved Kaplan-Meier Estimator in Survival Analysis Based on Partially Rank-Ordered Set Samples

**DOI:** 10.1155/2020/7827434

**Published:** 2020-05-29

**Authors:** Samane Nematolahi, Sahar Nazari, Zahra Shayan, Seyyed Mohammad Taghi Ayatollahi, Ali Amanati

**Affiliations:** ^1^Department of Biostatistics, Medical School, Shiraz University of Medical Sciences, Shiraz, Iran; ^2^Department of Medicine, University of Alberta, Edmonton, Canada; ^3^Professor Alborzi Clinical Microbiology Research Center, Shiraz University of Medical Sciences, Shiraz, Iran

## Abstract

This study presents a novel methodology to investigate the nonparametric estimation of a survival probability under random censoring time using the ranked observations from a Partially Rank-Ordered Set (PROS) sampling design and employs it in a hematological disorder study. The PROS sampling design has numerous applications in medicine, social sciences and ecology where the exact measurement of the sampling units is costly; however, sampling units can be ordered by using judgment ranking or available concomitant information. The general estimation methods are not directly applicable to the case where samples are from rank-based sampling designs, because the sampling units do not meet the identically distributed assumption. We derive asymptotic distribution of a Kaplan-Meier (KM) estimator under PROS sampling design. Finally, we compare the performance of the suggested estimators via several simulation studies and apply the proposed methods to a real data set. The results show that the proposed estimator under rank-based sampling designs outperforms its counterpart in a simple random sample (SRS).

## 1. Introduction

The idea of ranked set sampling (RSS) was introduced by McIntyre [1] for the first time. It can provide a more structural method for collecting the sample units. A generalization of RSS is the PROS sampling design. Both sampling methods are similar with a clear difference; in the PROS sampling design that we use in this paper, the ranker divides the sampling units into ranked subsets of prespecified sizes based on their partial ranks [2]. These sampling designs are techniques to obtain more representative samples from the underlying population where measurement of the units is costly and/or time-consuming. In such sampling designs, sampling units are ordered fairly accurately by using available auxiliary information which may be costly to some extent (see [3]).

After the PROS sampling design was introduced by Ozturk [4], many statisticians became interested in this rank-based sampling method. For example, Ozturk [5] and Frey [6] have relaxed the assumption concerning the prespecification of the number of subsets in each set. Nazari et al. [7] have developed nonparametric kernel density estimators using PROS data. Hatefi et al. [3] have applied PROS sampling in mixture modeling to estimate the age structures of short-lived fish species. Ozturk [8] have used the properties of PROS samples under multiple auxiliary information in the estimation of the population mean and total infinite population settings. Nazari et al. [9] have estimated the distribution function using PROS samples. Hatefi et al. [10] have studied the information and uncertainty structures of PROS data.

Currently, survival study is one of the important statistical tools for analyzing the data extracted from medical studies and social sciences. Presence of censoring observations is the distinction between survival analysis and other statistical analyses (see [11]). However, survival analyses are expensive due to the need of a large sample size and the potentially long follow-up duration [12]. For the sake of parsimony, we may consider the cost-effective sampling methods, in which only a small proportion of the available units is measured; however, they contain a portion of the information contributed by all of the units; for more information, see [13].

In this study, we develop the KM nonparametric estimator using the PROS sampling design. The KM estimator measures the probability that a person survives longer than a specific time, which is fundamental in survival analysis. We study the asymptotic properties of this new estimator and compare it with SRS and RSS counterparts. What distinguishes the present research from previous endeavors is that we employ the PROS sampling design for incomplete data containing censored observations, while all research on PROS sampling design has been concerned with the inference procedure for complete data. There are only a few results available when the researcher has incomplete data and the sampling design is based on RSS not PROS samples. For example, Yu and Tam [14] have considered maximum likelihood estimation of parameters of the log-normal distribution and have introduced a KM estimator for RSS. Zhang et al. [15] have used RSS for estimating the KM estimator of a reliability function with random right-censored data where the population distribution is unknown. Strzalkowska-Kominiak and Mahdizadeh [13] have proposed a KM estimator based on RSS when censored data are under random detection limit assumption. Mahdizadeh and Strzalkowska-Kominiak [16] have proposed a confidence interval for a distribution function when data are right-censored with random censoring time by applying RSS design.

In Section 2, we present some primary notes. In Section 3, we introduce the nonparametric KM estimator. In Section 4, we show the asymptotic normality of the KM estimator based on imperfect PROS sampling design. We compare the performance of the PROS KM estimator with respect to its SRS and RSS counterparts using simulation studies in Section 5. In addition, we illustrate our proposed method with a real example. We consider a dataset collected in Amir Medical Oncology Center, as our population in Section 6.

## 2. Necessary Background

### 2.1. Ranked Set Sampling

To obtain a RSS of size *nL*, with set size *n* and *L* cycles, from the underlying population, a set of *n* units is randomly selected from the population. The units are ranked via some mechanisms. Then, the unit that ranked as the smallest was selected for the final measurement. Another set of *n* units is drawn and ranked, and the unit ranked as the second smallest is selected for measurement. This process is continued until the unit ranked as the maximum is selected and measured. This is one cycle of the RSS procedure; the cycle can be repeated *L* times to generate RSS of size *nL* (see [17]).

### 2.2. Partially Rank-Ordered Set Sampling

In this section, we introduce the PROS sampling design and present the necessary notation. This sampling design is of the form *G*^∗∗^ design in Ozturk (see [4]). In order to extract a PROS sample of size *N* = *nL*, we choose a set size *s* = *nm* and a design parameter *D* = {*d*_1_, ⋯, *d*_*n*_}  that partitions the set {1, 2, ⋯, *s*}  into *n* mutually exclusive subsets *d*_*j*_ = {(*j* − 1)*m* + 1, ⋯, *jm*}, *j* = 1, ⋯, *n*. Sampling units are then assigned to the subsets *d*_*j*_, *j* = 1, ⋯, *n*, based on visual inspection, judgment ranking, or using a concomitant variable such that all units in the subset *d*_*j*_ are judged to have smaller ranks than all units in the subset *d*_*j*′_, when *j* < *j*′. A unit is then randomly selected from the subset *d*_1_ for full measurement and denoted by *X*_[*d*_1_]1_. Again, we randomly select a set containing *s*  units and assign them to *n*  subsets; after that, we randomly draw a member from subset *d*_2_ and denote it by  *X*_[*d*_2_]1_. These steps are continued until we randomly extract a unit from *d*_*n*_,  *X*_[*d*_*n*_]1_. These observations constitute one cycle of the PROS sampling design; after *L* repetitions of this process, we achieve a PROS sample of size *nL*, denoted by *X*_PROS_ = {*X*_[*d*_*j*_]*i*_, *i* = 1, ⋯, *L*, *j* = 1, ⋯, *n*}; for more details, see [9].

Table 1 presents a simple example of the construction of a PROS sample when *s* = 9, *n* = 3 , and *m* = 3, the cycle size is *L* = 2, and the design parameter is *D* = {*d*_1_, *d*_2_, *d*_3_} = {{1, 2, 3}, {4, 5, 6}, {7, 8, 9}}. Each set contains nine units assigned to three partially rank-ordered subsets. In this process, units in each subset have equal chance to take any place in the subset. One unit, in each set from the bold-faced subset, is randomly drawn and measured. The resulting PROS sample is denoted by {*X*_[*d*_*j*_]*i*_, *i* = 1, 2, *j* = 1, 2, 3}.

It should be noted that, if all members in the subset *d*_*j*_  have exactly smaller ranks than all members in *d*_*j*′_, *j* < *j*′, the PROS sampling design is perfect. Otherwise, we have an imperfect PROS sampling design. Suppose that *α* is a doubly stochastic matrix; we model the subsetting error probabilities in the imperfect PROS as follows (see [7] and [9]):

(1) $$\begin{array}{c} \alpha =\left[\begin{array}{ccc} \alpha_{d_{1},d_{1}} & \cdots & \alpha_{d_{1},d_{n}}\\ \vdots & \ddots & \vdots \\ \alpha_{d_{n},d_{1}} & \cdots & \alpha_{d_{n},d_{n}} \end{array}\right], \end{array}$$

where *α*_*d*_*j*_,*d*_*h*__ is the probability of assigning a unit into the subset *d*_*j*_ when it belongs to the subset  *d*_*h*_ with ∑_*h*=1_^*n*^*α*_*d*_*j*_,*d*_*h*__ = ∑_*j*=1_^*n*^*α*_*d*_*j*_,*d*_*h*__ = 1.

Throughout this paper, we use PROS_*α*_(*n*, *L*, *s*, *D*) as a symbol of an imperfect PROS sampling design with the design *D* = {*d*_*j*_, *j* = 1, ⋯, *n*}, where *α* represents a subsetting error probability matrix, *n*  shows the number of subsets, and *L*  and *s* exhibit the number of cycles and the set size, respectively. It should be pointed out that *m* = *s*/*n*.

SRS and RSS designs are special cases of the PROS sampling design when *s* = 1 and *s* = *n*, respectively. For a perfect PROS design, since *α*_*d*_j_,*d*_h__ = 0 for *h* ≠ *j*  and  *α*_*d*_j_,*d*_j__ = 1 for *j* = 1, ⋯, *n*, the subsetting error matrix is an identity matrix and the notation PROS_*I*_(*n*, *L*, *s*, *D*) can be used.

In this paper, the cumulative distribution function (CDF) of the studied variable in the population, CDF of  *X*_[*d*_*j*_]*i*_ for *i* = 1, ⋯, *L*, and CDF of the *r*th-order statistic among a simple random sample of size *s* are denoted by *F*, *F*_[*d*_*j*_]_, and *F*_(*r* : *s*)_, respectively. In addition, the corresponding probability density functions are represented by *f*, *f*_[*d*_*j*_]_, and *f*_(*r* : *s*)_.

## 3. Kaplan-Meier Estimator Based on PROS Sampling Design

**Definition 1 .**

Let *X*_1_, ⋯, *X*_*n*_ ~ *F* and *C*_1_, ⋯, *C*_*n*_ ~ *G* be two independent random variables where we observe  *Y*_*i*_ = min{*X*_*i*_, *C*_*i*_} ~ *H* and *δ*_*i*_ = 1{*X*_*i*_ ≤ *C*_*i*_} be the indicator variable which specifies the event/censored status. The KM estimator defined as

(2) $$\begin{array}{c} 1-{\hat{F}}_{\text{SRS}}\left(t\right)=\overset{n}{\underset{i=1}{\prod }}{\left(1-\frac{\delta_{\left[i\right]}}{n-i+1}\right)}^{1\left\{Y_{\left(i\right)}\leq t\right\}}, \end{array}$$

where *Y*_(1)_, ⋯, *Y*_(*n*)_ are ordered values of the simple random sample (SRS) with related *δ*_[1]_, ⋯, *δ*_[*n*]_ values; see [18] for more information.

Based on the above Definition 1 and Definition 1 in [9], we estimate the KM estimator based on the imperfect PROS sampling design PROS_*α*_(*n*, *L*, *s*, *D*).

The KM estimator based on the PROS_*α*_(*n*, *L*, *s*, *D*) sample,  X_PROS_, defined as

(3) $$\begin{array}{c} 1-{\hat{F}}_{\text{PROS}}\left(t\right)=\frac{1}{n}\overset{n}{\underset{j=1}{\sum }}\left(1-{\hat{F}}_{\left[d_{j}\right]}\left(t\right)\right), \end{array}$$

where $1-{\hat{F}}_{\left[d_{j}\right]}\;$ is the KM estimator based on the independent and identically distributed (SRS) {*Y*_[*d*_*j*_]1_, *Y*_[*d*_*j*_]2_, ⋯, *Y*_[*d*_*j*_]*L*_}, defined as

(4) $$\begin{array}{c} 1-{\hat{F}}_{\left[d_{j}\right]}\left(t\right)=\overset{L}{\underset{k=1}{\prod }}{\left(1-\frac{\delta_{\left[d_{j}\right]k}^{*}}{L-k+1}\right)}^{1\left\{Y_{\left[d_{j}\right]k}^{*}\leq t\right\}}, \end{array}$$

where  *Y*_[*d*_*j*_]1_^∗^, ⋯, *Y*_[*d*_*j*_]*L*_^∗^  are ordered values of  *Y*_[*d*_*j*_]1_, ⋯, *Y*_[*d*_*j*_]*L*_ and *δ*_[*d*_*j*_]*k*_^∗^ values are related to *Y*_[*d*_*j*_]*k*_^∗^ values for *k* = 1, ⋯, *L*.

## 4. Asymptotic Properties

In this section, we study the behavior of the nonparametric KM estimator in large samples based on the imperfect PROS sampling design. The asymptotic properties of the KM estimator under the SRS were widely available in the literature survey [19–21].

We demonstrate that no stronger assumptions are needed while using the imperfect PROS-based KM estimator. At first, we introduce the following lemma, which is a straight result of Lemma 2.1 in Stute and Wang [18].

**Lemma 1 .**

Suppose *X* ~ *F* and *C* ~ *G* are two independent random variables. In addition, let *X*_[*d*_*j*_]*i*_ ~ *F*_[*d*_*j*_]_ be the PROS sample from subset *d*_*j*_ in the *i*th cycle and *C*_[*d*_*j*_]*i*_ be the corresponding censored time.

Set  *H*_[*d*_*j*_]_(*t*) = *P*(min(*X*_[*d*_*j*_]*i*_, *C*_[*d*_*j*_]*i*_) ≤ *t*), then we have

(5) $$\begin{array}{c} 1-H_{\left[d_{j}\right]}\left(t\right)=\left(1-F_{\left[d_{j}\right]}\left(t\right)\right)\left(1-G\left(t\right)\right). \end{array}$$

**Proof**

See Appendix A.

Due to the expressed lemma, we can define

(6) $$\begin{array}{c} {\tilde{H}}_{\left[d_{j}\right]}^{0}\left(z\right)=\int_{-\infty }^{z}\left(1-F_{\left[d_{j}\right]}\left(y\right)\right)G\left(dy\right),\\ {\tilde{H}}_{\left[d_{j}\right]}^{1}\left(z\right)=\int_{-\infty }^{z}\left(1-G\left(y-\right)\right)F_{\left[d_{j}\right]}\left(dy\right). \end{array}$$

We also set

(7) $$\begin{array}{c} \gamma_{0d_{j}}\left(X\right)=\exp\left\{\int_{-\infty }^{x}\frac{{\tilde{H}}_{\left[d_{j}\right]}^{0}\left(dz\right)}{1-H_{\left[d_{j}\right]}\left(z\right)}\right\}. \end{array}$$

Let *φ*(*w*) be a score function

(8) $$\begin{array}{c} \gamma_{1d_{j}}\left(X\right)=\frac{1}{1-H_{\left[d_{j}\right]}\left(x\right)}\int 1\left\{x<w\right\}\varphi \left(w\right)\gamma_{0d_{j}}\left(w\right){\tilde{H}}_{\left[d_{j}\right]}^{1}\left(dw\right),\\ \gamma_{2d_{j}}\left(X\right)=\iint \frac{1\left\{v<x,v<w\right\}\varphi \left(w\right)\gamma_{0d_{j}}\left(w\right)}{{\left(1-H_{\left[d_{j}\right]}\left(v\right)\right)}^{2}}{\tilde{H}}_{\left[d_{j}\right]}^{0}\left(dv\right){\tilde{H}}_{\left[d_{j}\right]}^{1}\left(dw\right). \end{array}$$

Now, we present Theorem 1.

**Theorem 1 .**

Assume *F* and *G*  are continuous and

(9) $$\begin{array}{c} \int \varphi^{2}\left(x\right)\gamma_{0}^{2}\left(x\right){\tilde{H}}^{1}\left(dx\right)<\infty , \end{array}$$

(10) $$\begin{array}{c} \int \left|\varphi \left(x\right)\right|{\left(\int_{-\infty }^{x}\frac{G\left(dz\right)}{\left(1-H\left(z\right)\right)\left(1-G\left(z\right)\right)}\right)}^{1/2}F\left(dx\right)<\infty , \end{array}$$

where

(11) $$\begin{array}{c} {\tilde{H}}^{0}\left(z\right)=P\left(X\leq z,\delta =0\right)=\int_{-\infty }^{z}\left(1-F\left(y\right)\right)G\left(dy\right),z\in \mathbb{R},\\ {\tilde{H}}^{1}\left(z\right)=P\left(X\leq z,\delta =1\right)=\int_{-\infty }^{z}\left(1-G\left(y\right)\right)F\left(dy\right),z\in \mathbb{R}. \end{array}$$

Also, set

(12) $$\begin{array}{c} \gamma_{0}\left(x\right)=\exp\left\{\int_{-\infty }^{x}\frac{{\tilde{H}}^{0}\left(dz\right)}{1-H\left(z\right)}\right\},\\ \gamma_{1}\left(x\right)=\frac{1}{1-H\left(x\right)}\int 1\left\{x<w\right\}\varphi \left(w\right)\gamma_{0}\left(w\right){\tilde{H}}^{1}\left(dw\right),\\ \gamma_{2}\left(x\right)=\int \frac{\int 1\left\{v<x,v<w\right\}\varphi \left(w\right)\gamma_{0}\left(w\right)}{{\left(1-H\left(v\right)\right)}^{2}}{\tilde{H}}^{0}\left(dv\right){\tilde{H}}^{1}\left(dw\right). \end{array}$$

As  *L*⟶∞ and  *N* = *nL*, we have

(13) $$\begin{array}{c} \sqrt{N}\int \varphi \left(x\right)\left({\hat{F}}_{\text{PROS}}\left(dx\right)-F\left(dx\right)\right)\sim N\left(0,\sigma_{n}^{2}\right), \end{array}$$

where

(14) $$\begin{array}{c} \sigma_{n}^{2}=\frac{1}{n}\overset{n}{\underset{j=1}{\sum }}\mathrm{var}\left[\varphi_{\left(Y_{\left[dj\right]}\right)}\;\gamma_{0dj}\left(Y_{\left[dj\right]}\right)\delta_{\left[dj\right]}+\gamma_{1dj}\left(Y_{\left[dj\right]}\right)\left(1-\delta_{\left[dj\right]}\right)-\gamma_{2dj}\left(Y_{\left[dj\right]}\right)\right]. \end{array}$$

**Proof**

In view of the equivalent theorem in SRS sampling design [21], it suffices to show that, for every *j* = 1, ⋯, *n*,

(15) $$\begin{array}{c} \int \varphi^{2}\left(x\right){\gamma^{2}}_{0dj}\left(x\right){{\tilde{H}}^{1}}_{\left[dj\right]}\left(dx\right)<\infty , \end{array}$$

(16) $$\begin{array}{c} \int \left|\varphi \left(x\right)\right|{\left(\int_{-\infty }^{x}\frac{G\left(dz\right)}{\left(1-H_{\left[d_{j}\right]}\left(z\right)\right)\left(1-G\left(z\right)\right)}\right)}^{1/2}\;F_{\left[d_{j}\right]}\left(dx\right)<\infty . \end{array}$$

As to equation (15), under continuity of *F* and  *G* and *γ*_0*dj*_(*X*) = (1 − *G*(*x*))^−1^, we also have

(17) $$\begin{array}{c} {\tilde{H}}_{\left[d_{j}\right]}^{1}\left(dx\right)=\frac{d\left({\tilde{H}}_{\left[d_{j}\right]}^{1}\left(x\right)\right)}{dx}=\frac{d\left(\int_{-\infty }^{x}\left(1-G\left(y\right)\right)F_{\left[d_{j}\right]}\left(dy\right)\right)}{dx}=\left(1-G\left(x\right)\right)F_{\left[d_{j}\right]}\left(dx\right). \end{array}$$

Under the continuity of *F*, there exists a density *f*. We have *F*(*dx*) = (1/*n*)∑_*j*=1_^*n*^*F*_[*d*_*j*_]_(*dx*); hence, *nF*(*dx*) = ∑_*j*=1_^*n*^*F*_[*d*_*j*_]_(*dx*).

By using the above relationship,

(18) $$\begin{array}{c} \int \varphi^{2}\left(x\right){\gamma^{2}}_{0d_{j}}\left(x\right){\tilde{H}}_{\left[d_{j}\right]}^{1}\left(dx\right)=\int \frac{\varphi^{2}\left(x\right)}{{\left(1-G\left(x\right)\right)}^{2}}\left(1-G\left(x\right)\right)F_{\left[d_{j}\right]}\left(dx\right)=\int \frac{\varphi^{2}\left(x\right)}{\left(1-G\left(x\right)\right)}F_{\left[d_{j}\right]}\left(dx\right)\leq \overset{n}{\underset{j=1}{\sum }}\int \frac{\varphi^{2}\left(x\right)}{\left(1-G\left(x\right)\right)}F_{\left[d_{j}\right]}\left(dx\right)=n\int \frac{\varphi^{2}\left(x\right)}{\left(1-G\left(x\right)\right)}F\left(dx\right)<\infty . \end{array}$$

By equation (9), this phrase is finite, so we prove equation (15).

To prove that (16) holds, we have to determine a lower bound for 1 − *F*_[*d*_*j*_]_(*z*).

We know that

(19) $$\begin{array}{c} \overset{s}{\underset{i=s-u+1}{\sum }}\left(\begin{array}{c} s\\ s-i \end{array}\right){\left(1-F\left(z\right)\right)}^{i-\left(s-u+1\right)}{\left(F\left(z\right)\right)}^{s-i}=\overset{u-1}{\underset{i=0}{\sum }}\left(\begin{array}{c} s\\ u-1-i \end{array}\right){\left(1-F\left(z\right)\right)}^{i}{\left(F\left(z\right)\right)}^{u-1-i}, \end{array}$$

for  *i* ≤ *u* − 1, we have

(20) $$\begin{array}{c} \left(\begin{array}{c} s\\ u-1-i \end{array}\right)\geq \left(\begin{array}{c} u-1\\ i \end{array}\right),\\ 1-F_{\left(u:s\right)}\left(z\right)=\overset{s}{\underset{i=s-u+1}{\sum }}\left(\begin{array}{c} s\\ i \end{array}\right){\left(1-F\left(z\right)\right)}^{i}{\left(F\left(z\right)\right)}^{s-i}={\left(1-F\left(z\right)\right)}^{s-u+1}\overset{s}{\underset{i=s-u+1}{\sum }}\left(\begin{array}{c} s\\ s-i \end{array}\right){\left(1-F\left(z\right)\right)}^{i-\left(s-u+1\right)}{\left(F\left(z\right)\right)}^{s-i}={\left(1-F\left(z\right)\right)}^{s-u+1}\overset{u-1}{\underset{i=0}{\sum }}\left(\begin{array}{c} s\\ u-1-i \end{array}\right){\left(1-F\left(z\right)\right)}^{i}{\left(F\left(z\right)\right)}^{u-1-i}\geq {\left(1-F\left(z\right)\right)}^{s-u+1}\overset{u-1}{\underset{i=0}{\sum }}\left(\begin{array}{c} u-1\\ i \end{array}\right){\left(1-F\left(z\right)\right)}^{i}{\left(F\left(z\right)\right)}^{u-1-i}={\left(1-F\left(z\right)\right)}^{s-u+1},\Rightarrow 1-F_{\left(u:s\right)}\left(z\right)\geq {\left(1-F\left(z\right)\right)}^{s-u+1}. \end{array}$$

Therefore, we have

(21) $$\begin{array}{c} 1-F_{\left[d_{j}\right]}\left(z\right)=\frac{1}{m}\overset{n}{\underset{h=1}{\sum }}\underset{u\in d_{h}}{\sum }\alpha_{d_{j},d_{h}}\left(1-F_{\left(u:s\right)}\left(z\right)\right)\geq \frac{1}{m}\overset{n}{\underset{h=1}{\sum }}\underset{u\in d_{h}}{\sum }\alpha_{d_{j},d_{h}}{\left(1-F\left(z\right)\right)}^{s-u+1}. \end{array}$$

We know

(22) $$\begin{array}{c} f_{\left[d_{j}\right]}\left(x\right)=\frac{1}{m}\overset{n}{\underset{h=1}{\sum }}\underset{u\in d_{h}}{\sum }\alpha_{d_{j},d_{h}}f_{\left(u:s\right)}\left(x\right)=\frac{1}{m}\overset{n}{\underset{h=1}{\sum }}\underset{u\in d_{h}}{\sum }\alpha_{d_{j},d_{h}}\frac{s!}{\left(u-1\right)!\left(s-u\right)!}f\left(x\right){F\left(x\right)}^{u-1}{\left(1-F\left(x\right)\right)}^{s-u}\leq \frac{1}{m}\overset{n}{\underset{h=1}{\sum }}\underset{u\in d_{h}}{\sum }\alpha_{d_{j},d_{h}}\frac{s!}{\left(u-1\right)!\left(s-u\right)!}f\left(x\right){\left(1-F\left(x\right)\right)}^{s-u}. \end{array}$$

Also,

(23) $$\begin{array}{c} z\leq x\Rightarrow {\left(1-F\left(z\right)\right)}^{s-u}\geq {\left(1-F\left(x\right)\right)}^{s-u}, \end{array}$$

so,

(24) $$\begin{array}{c} \frac{1}{m}\overset{n}{\underset{h=1}{\sum }}\underset{u\in d_{h}}{\sum }\alpha_{d_{j},d_{h}}{\left(1-F\left(z\right)\right)}^{s-u}\geq \frac{1}{m}\overset{n}{\underset{h=1}{\sum }}\underset{u\in d_{h}}{\sum }\alpha_{d_{j},d_{h}}{\left(1-F\left(x\right)\right)}^{s-u}. \end{array}$$

Then,

(25) $$\begin{array}{c} {\left(\frac{1}{m}\overset{n}{\underset{h=1}{\sum }}\underset{u\in d_{h}}{\sum }\alpha_{d_{j},d_{h}}{\left(1-F\left(z\right)\right)}^{s-u}\right)}^{-\left(1/2\right)}\leq {\left(\frac{1}{m}\overset{n}{\underset{h=1}{\sum }}\underset{u\in d_{h}}{\sum }\alpha_{d_{j},d_{h}}{\left(1-F\left(x\right)\right)}^{s-u}\right)}^{-\left(1/2\right)}. \end{array}$$

Based on Lemma 1 and the above equations

(26) $$\begin{array}{c} \int \left|\varphi \left(x\right)\right|{\left(\int_{-\infty }^{x}\frac{G\left(dz\right)}{\left(1-H_{\left[d_{j}\right]}\left(z\right)\right)\left(1-G\left(z\right)\right)}\right)}^{1/2}F_{\left[d_{j}\right]}\left(dx\right)=\int \left|\varphi \left(x\right)\right|{\left(\int_{-\infty }^{x}\frac{G\left(dz\right)}{\left(1-F_{\left[d_{j}\right]}\left(z\right)\right){\left(1-G\left(z\right)\right)}^{2}}\right)}^{1/2}f_{\left[d_{j}\right]}\left(x\right)dx\leq \int \left|\varphi \left(x\right)\right|{\left(\int_{-\infty }^{x}\frac{G\left(dz\right)}{\left(\left(1/m\right)\sum_{h=1}^{n}\sum_{u\in d_{h}}\alpha_{d_{j},d_{h}}{\left(1-F\left(z\right)\right)}^{s-u+1}\right){\left(1-G\left(z\right)\right)}^{2}}\right)}^{1/2}\frac{1}{m}\overset{n}{\underset{h=1}{\sum }}\underset{u\in d_{h}}{\sum }\alpha_{d_{j},d_{h}}\frac{s!}{\left(u-1\right)!\left(s-u\right)!}f\left(x\right){\left(1-F\left(x\right)\right)}^{s-u}dx=\int \;|\;\varphi \left(x\right)\;|\;{\left(\int_{-\infty }^{x}\frac{G\left(dz\right)}{\left(1-F\left(z\right)\right){\left(1-G\left(z\right)\right)}^{2}}\times \frac{1}{\left(1/m\right)\sum_{h=1}^{n}\sum_{u\in d_{h}}\alpha_{d_{j},d_{h}}{\left(1-F\left(z\right)\right)}^{s-u}}\right)}^{1/2}\frac{1}{m}\overset{n}{\underset{h=1}{\sum }}\underset{u\in d_{h}}{\sum }\alpha_{d_{j},d_{h}}\frac{s!}{\left(u-1\right)!\left(s-u\right)!}{\left(1-F\left(x\right)\right)}^{s-u}F\left(dx\right)\leq \int \;|\;\varphi \left(x\right)\;|\;{\left(\int_{-\infty }^{x}\frac{G\left(dz\right)}{\left(1-H\left(z\right)\right)\left(1-G\left(z\right)\right)}\right)}^{1/2}{\left(\frac{1}{m}\overset{n}{\underset{h=1}{\sum }}\underset{u\in d_{h}}{\sum }\alpha_{d_{j},d_{h}}{\left(1-F\left(x\right)\right)}^{s-u}\right)}^{-\left(1/2\right)}\left(\frac{1}{m}\overset{n}{\underset{h=1}{\sum }}\underset{u\in d_{h}}{\sum }\alpha_{d_{j},d_{h}}\frac{s!}{\left(u-1\right)!\left(s-u\right)!}{\left(1-F\left(x\right)\right)}^{s-u}\right)F\left(dx\right)\leq \int \;|\;\varphi \left(x\right)\;|\;{\left(\int_{-\infty }^{x}\frac{G\left(dz\right)}{\left(1-H\left(z\right)\right)\left(1-G\left(z\right)\right)}\right)}^{1/2}{\left(\frac{1}{m}\overset{n}{\underset{h=1}{\sum }}\underset{u\in d_{h}}{\sum }\alpha_{d_{j},d_{h}}{\left(1-F\left(x\right)\right)}^{s-u}\right)}^{-\left(1/2\right)}\left(\frac{C}{m}\overset{n}{\underset{h=1}{\sum }}\underset{u\in d_{h}}{\sum }\alpha_{d_{j},d_{h}}{\left(1-F\left(x\right)\right)}^{s-u}\right)F\left(dx\right)=C\int \;|\;\varphi \left(x\right)\;|\;{\left(\int_{-\infty }^{x}\frac{G\left(dz\right)}{\left(1-H\left(z\right)\right)\left(1-G\left(z\right)\right)}\right)}^{1/2}{\left(\frac{1}{m}\overset{n}{\underset{h=1}{\sum }}\underset{u\in d_{h}}{\sum }\alpha_{d_{j},d_{h}}{\left(1-F\left(x\right)\right)}^{s-u}\right)}^{1/2}F\left(dx\right). \end{array}$$

We define constant *C* as

(27) $$\begin{array}{c} C=\max_{u=1,\cdots ,s}\left\{\frac{s!}{\left(u-1\right)!\left(s-u\right)!}\;\right\}. \end{array}$$

Because  *s* − *u* ≥ 0, we have

(28) $$\begin{array}{c} \frac{1}{m}\overset{n}{\underset{h=1}{\sum }}\underset{u\in d_{h}}{\sum }\alpha_{d_{j},d_{h}}{\left(1-F\left(x\right)\right)}^{s-u}\leq \frac{1}{m}\overset{n}{\underset{h=1}{\sum }}\underset{u\in d_{h}}{\sum }\alpha_{d_{j},d_{h}}=1, \end{array}$$

so (26) is smaller than

(29) $$\begin{array}{c} C\int \left|\varphi \left(x\right)\right|{\left(\int_{-\infty }^{x}\frac{G\left(dz\right)}{\left(1-H\left(z\right)\right)\left(1-G\left(z\right)\right)}\right)}^{1/2}F\left(dx\right). \end{array}$$

In view of equation (10), this equation is finite, and this completes the proof.

It should be noted that Theorem 1 has been proven only for the imperfect model, which has already been described in Section 2.2, and this model is not completely general.

## 5. Simulation Study

In this section, we compare the performance of the KM estimator of survival function under the PROS sampling design relative to its SRS and RSS counterparts.

To do so, we considered two situations in which the original random variables were generated from an exponential distribution with mean 1 (model A) and standard log-normal distribution with mean 1.649 (model B). The censored variables in the two cases are supposed to have an exponential distribution; a common rate of exponential distribution was determined when the desired censoring level was prespecified. In all simulation scenarios *s* = *nm*  and the set size for the RSS sampling design is *n*. The algorithm of the simulation study is explained in Appendix B.

By using distribution theory, if *D* and *E* are independent and distributed exponentially with means *θ*_1_  and *θ*_2_, respectively, then *P*(*D* ≤ *E*) = *θ*_2_/(*θ*_1_ + *θ*_2_). On the other hand, *P*(*D* ≤ *E*) = 1 − *p*. Setting the values of the censoring level (*p*) and *θ*_1_ = 1 in these equations, we can find the appropriate value of the exponential rate in model A. Given the fact that there is no such expression for model B, we found the exponential common rate for the censoring variable by trial and error, although one can easily solve this problem numerically by using software like R. The values of the exponential rate were equal to 0.013 and 0.190 and led to censoring levels of 0.1 and 0.6, respectively.

For each combination of sample sizes *N* = 30, 120, and 240 and the mentioned censoring levels 0.1 and 0.6, 5000 samples were generated under the SRS, RSS, and PROS sampling designs. For different values of *n*, *m*, and *L* and the misplacement probabilities *α*_*d*_*i*_,*d*_*i*__ = *α*_0_ and *α*_*d*_*i*_,*d*_*j*__ = (1 − *α*_0_)/(*n* − 1) for  *i* ≠ *j*, the values of the mean squared error (MSE) were computed for the three estimators from each sample when *α*_0_ = 0, 0.5, 0.7, and 1.

### 5.1. Comparing the Kaplan-Meier Estimators

We compare the performance of the KM estimators of the survival function between the studied sampling designs. The efficiency of the PROS estimation with respect to its SRS and RSS counterparts, at the point *t*, is defined as

(30) $$\begin{array}{c} \text{RP}=\frac{\text{MSE}\left(1-{\hat{F}}_{\text{RSS}}\left(t\right)\right)}{\text{MSE}\left(1-{\hat{F}}_{\text{PROS}}\left(t\right)\right)},\text{SP}=\frac{\text{MSE}\left(1-{\hat{F}}_{\text{SRS}}\left(t\right)\right)}{\text{MSE}\left(1-{\hat{F}}_{\text{PROS}}\left(t\right)\right)}, \end{array}$$

where $1-{\hat{F}}_{\text{PROS}}\left(t\right)$, $1-{\hat{F}}_{\text{RSS}}\left(t\right)$, and $1-{\hat{F}}_{\text{SRS}}\left(t\right)$ are the KM estimators of the survival function at point *t* based on PROS, RSS, and SRS sampling designs, respectively.

Note that $\text{ MSE}\left(1-{\hat{F}}_{\text{PROS}}\left(t\right)\right)=E{\left[F\left(t\right)-{\hat{F}}_{\text{PROS}}\left(t\right)\right]}^{2}$. $\text{MSE}\left(1-{\hat{F}}_{\text{RSS}}\left(t\right)\right)$ and $\text{MSE}\left(1-{\hat{F}}_{\text{SRS}}\left(t\right)\right)$ are similarly defined. Also, *t* = *F*^−1^(*q*) for a fixed percentile  *q* ∈ (0, 1), and *F*^−1^(.) is the inverse of the underlying distribution function. The values of RP and SP calculate for *m* = 3 and *n* = 3 and 5 in both models when we consider *q* = 0.10, 0.25, 0.50, 0.75, and 0.90. Because of the large volume of output and similar results in both models, we only report the results for model A in this article.

In the literature, the sample sizes in the PROS and RSS designs were similar but they have used a much smaller set size for RSS sampling design than for PROS. However, simulation studies that are not presented here show that the RSS-based estimator may performs better than the one using the PROS sampling design under the same sample size and the same set size.

**Figure 1:**
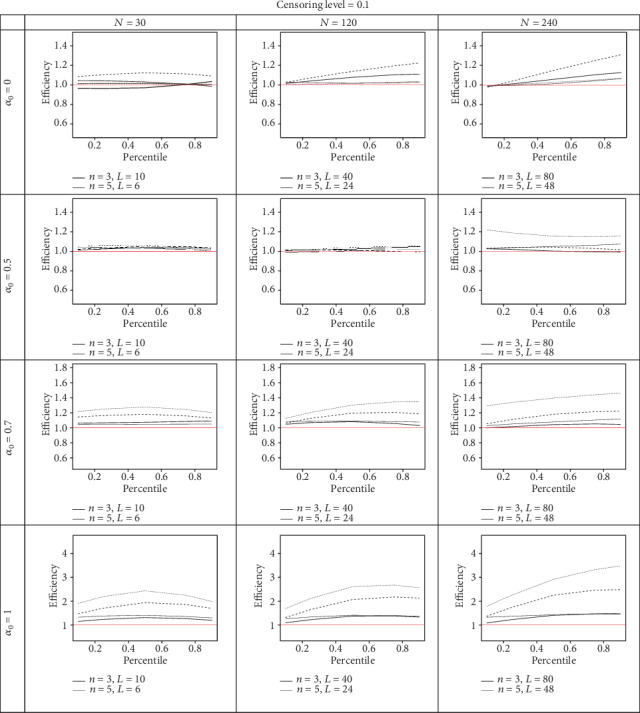
The efficiency of the KM estimator based on PROS with respect to RSS (solid line) and SRS (dashed line) counterparts at different percentiles.

**Figure 2:**
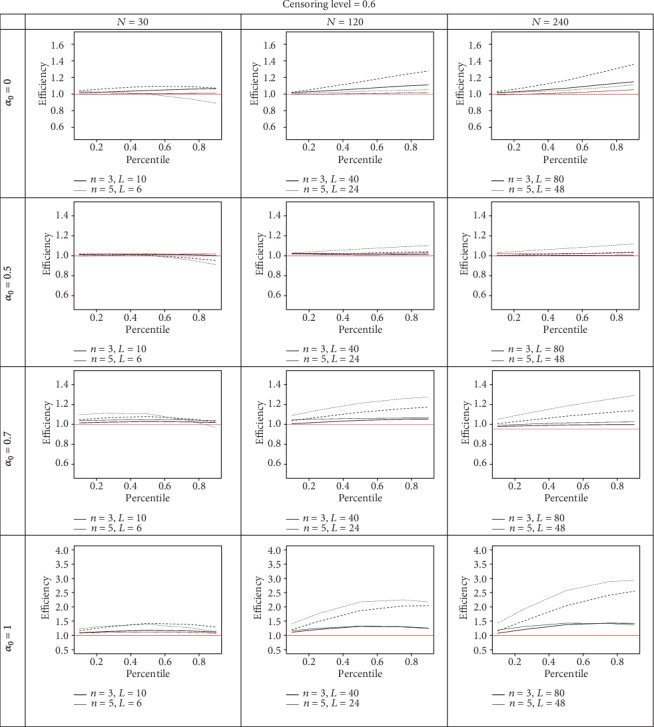
The efficiency of KM estimator based on PROS with respect to RSS (solid line) and SRS (dashed line) counterparts at different percentiles.

As shown in Figures 1 and 2, in model A, the KM estimator based on the PROS sampling design in most cases is more efficient than the KM estimator based on the RSS and SRS sampling designs with similar sample sizes. The best performance of the PROS design over the SRS and RSS designs happens when the ranking errors are small or zero, i.e., when *α*_0_ = 0.7 and 1. The efficiency of the KM estimator based on PROS relative to SRS is as good as or higher than the efficiency of the KM estimator based on the PROS relative to the RSS procedure, regardless of the censoring level and ranking error. Assuming a fixed sample size and censoring level, by increasing the *n* for large values of *α*_0_, the efficiency of the KM estimator based on the PROS sampling design is enhanced. It should be noted that in an imperfect PROS sampling design (*α*_0_ = 0), the efficiency reduced as *n* increased.

We can conclude that increasing the level of censorship in a smaller sample size leads to a reduction in efficiency in both models, but for a larger sample size, this rarely happens; in other words, the level of censored data in the smaller sample size has a greater impact on the performance of the PROS sampling design compared to the that in the larger sample size.

We conclude that, regardless of the censoring level and ranking error, increasing the sample size leads to increased efficiency. The perfect PROS KM estimator performs three times more efficiently than the SRS KM estimator in several simulation scenarios. It is worth noting that RP might decrease when one considers the same set size in the PROS and RSS designs with similar sample sizes. In all figures, we consider *m* = 3 for the PROS design.

In addition, we compared these three sampling methods using a mean integrated squared error (MISE) indicator, defined as

(31) $$\begin{array}{c} \text{MISE}=\int_{-\infty }^{+\infty }E{\left\{{\hat{F}}_{n}\left(t\right)-F\left(t\right)\right\}}^{2}dF\left(t\right). \end{array}$$

From Table 2, we can conclude that most of the time, PROS has less MISE than the RSS and SRS sampling methods with similar sample sizes, especially for a large *α*_0_. In addition, we observe that as the level of censored data increases, the amount of the MISE value increases as well in both models. It should be mentioned that in the low level of censorship, the log-normally distributed (model B) has lower MISE than the exponentially distributed (model A), but at the high level of censorship, model B has larger MISE than model A, for the same values of *n* and *m* and the subsetting probabilities *α*_*d*_*i*_,*d*_*j*__. As we expect, increasing the sample size reduces the MISE.

The results show that when *α*_0_ = 0.5, 0.7, and 1 in a smaller sample size with a low percentage of censored data, the larger *n* leads to the smaller MISE of the estimators, but with a high percentage of censored data, the MISE value increases as *n* increases. However, in larger sample sizes, the MISE of the estimator decreases as the *n* goes up in all censoring levels.

In Table 2, as the misplacement probabilities decrease, the superiority of the PROS estimator compared to the RSS and SRS estimators becomes more obvious. The MISE values of the KM estimator derived from perfect PROS and perfect RSS sampling designs are smaller than those in imperfect methods. Note that the KM estimator based on the SRS sampling design has a smaller MISE value than the one based on the imperfect rank-based sampling designs for some cases in small sample sizes and high censorship percentage.

Note that the RSS KM estimator can have a lower MISE than the PROS one, when we consider a similar set size and fixed sample size.

## 6. Real Data Application

In this section, we use the information of children under 18 years of age with nonhematological disorders such as Beta-Thalassemia and Idiopathic Thrombocytopenic Purpura (ITP) and children with hematological malignancies including various types of lymphoma and Acute Lymphocytic Leukemia (ALL), registered in the Amir Medical Oncology Center during May 2014 to August 2017. The dataset contains the survival information of 61 patients. We provide KM estimates of *Y* which is the survival time (in months) as the variable of interest by using *Z* which is the white blood cells as the concomitant variable, which are used for ranking purpose. The correlation coefficient between *Z* and *Y* is 0.455 and is significant (*p* value = 0.0001); also, we should add that 50.8% of people are censored. We considered the perfect PROS and RSS sampling designs. In order to estimate the KM estimator of survival time, we regarded this data set as a target population and extract PROS, RSS, and SRS samples (with replacement) of size *N* = *nL* from the population. We considered design parameter  *D* = {*d*_1_, *d*_2_, *d*_3_, *d*_4_, *d*_5_}. At the first step, we randomly selected *nm* = 15 patients from the target population and then partitioned these patients into subsets *d*_1_, *d*_2_, *d*_3_, *d*_4_, and *d*_5_ based on their WBC values. At the next step, we randomly selected a unit from subset *d*_1_ and observed its survival time. Again, we randomly selected 15 patients and assigned them to *d*_1_, *d*_2_, *d*_3_, *d*_4_, and *d*_5_ and randomly drew a member from subset *d*_2_ and repeated these steps until we selected a unit from subset *d*_5_; these observations constitute one cycle of PROS; in this real data, we considered 3 cycles, and finally, we have 15 survival time observations from patients.

In RSS, we randomly selected 5 patients from the target population and ranked them based on their WBC values, then we selected the patient with the smallest WBC and observed its survival time. This procedure continued until the survival time of the 5th ranked unit in the 5th set of units measured. These 5 observations constitute one cycle of RSS; in this example, we considered 3 cycles, and finally, we observed the survival time of 15 patients.

**Figure 3:**
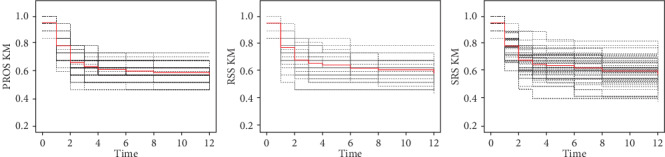
The KM estimates in different time for *n* = 5, *L* = 3, and *m* = 3 under 50 PROS, RSS, and SRS samples. The red solid line shows the mean of KM estimators.

For each sampling design, the KM estimator was calculated in different time points. Then, this process was repeated *M* times. We took (*n*, *m*, *L*, *M*) = (5, 3, 3, 50). These 50 KM charts under the three sampling designs are shown in Figure 3. Figure 3 shows that the variation of the KM estimators in each fixed time under the PROS sampling design is less than the variation of the RSS and SRS counterparts. We conclude that in this real data, the PROS estimate performs better than the RSS and SRS designs. We uploaded the raw data as a supplementary material (available here).

## 7. Summary and Concluding Remarks

In numerous medical fields, the exact measurement of the desired variable is expensive or time-consuming. Rank-based sampling designs such as PROS can help overcome this difficulty by ranking a small number of sampling units based on a concomitant variable. These sampling designs can be used to obtain samples that are more informative and also result in more accurate inference about the parameters of interest.

In this paper, we considered the problem of the KM estimator that is a proper and commonly used technique in survival analysis associated with an imperfect PROS sampling design. PROS is a new sampling design that avoids ranking all units in a given set. Furthermore, we developed asymptotic distributional properties of the new KM estimator based on a proposed sampling method. We showed how well this estimator performs in comparison with its RSS and SRS counterparts. The simulation results recommend that under both perfect and imperfect subsetting assumptions, the efficiency of the estimator based on the PROS sampling design is higher than the efficiency of the estimator based on the two other sampling methods with the same sample sizes. It is noteworthy that, by increasing the set size in RSS while keeping the sample size fixed in both designs, the RSS KM estimator can have smaller values of MSE than the PROS one. Finally, we applied all the introduced sampling designs to a real data set. We believe that it would be appealing to apply the proposed methodology to useful statistical models, for example, a Cox regression model for analyzing time to event data that is applicable to the majority of medical fields.

Finally, we will recommend the use of recently proposed sampling designs to extend this study, for example, even order ranked set sampling (EORSS) [22] and quartile pair ranked set sampling (QPRSS) [23] designs that have recently received attention by some researchers.

## Figures and Tables

**Table 1:** An example of a Partially Rank-Ordered Set sample.

Cycle	Set	Subset	Observation
1	*S*_1_	*D*_1_ = {**d**_1_, *d*_2_, *d*_3_} = {{1, 2, 3}, {4, 5, 6}, {7, 8, 9}}	*X*_[*d*1]1_
	*S*_2_	*D*_2_ = {*d*_1_, **d**_2_, *d*_3_} = {{1, 2, 3}, {4, 5, 6}, {7, 8, 9}}	*X*_[*d*2]1_
	*S*_3_	*D*_3_ = {*d*_1_, *d*_2_, **d**_3_} = {{1, 2, 3}, {4, 5, 6}, {7, 8, 9}}	*X*_[*d*3]1_

2	*S*_1_	*D*_1_ = {**d**_1_, *d*_2_, *d*_3_} = {{1, 2, 3}, {4, 5, 6}, {7, 8, 9}}	*X*_[*d*1]2_
	*S*_2_	*D*_2_ = {*d*_1_, **d**_2_, *d*_3_} = {{1, 2, 3}, {4, 5, 6}, {7, 8, 9}}	*X*_[*d*2]2_
	*S*_3_	*D*_3_ = {*d*_1_, *d*_2_, **d**_3_} = {{1, 2, 3}, {4, 5, 6}, {7, 8, 9}}	*X*_[*d*3]2_

**Table 2:** Estimated MISE of Kaplan Meier estimator, *N* = 30, 120, and 240.

		Censoring level	*α*_0_ = 0			*α*_0_ = 0.5			*α*_0_ = 0.7			*α*_0_ = 1		
			PROS	RSS	SRS	PROS	RSS	SRS	PROS	RSS	SRS	PROS	RSS	SRS
Model A	*N* = 30(*n* = 3, *L* = 10)	0.1	0.0062	0.0065	0.0071	0.0066	0.0069	0.0069	0.0059	0.0064	0.0071	0.0037	0.0048	0.0070
		0.6	0.0620	0.0653	0.0664	0.0672	0.0679	0.0653	0.0628	0.0644	0.0655	0.0492	0.0563	0.0661
	*N* = 30(*n* = 5, *L* = 6)	0.1	0.0069	0.0068	0.0071	0.0065	0.0068	0.0069	0.0055	0.0058	0.0071	0.0030	0.0042	0.0070
		0.6	0.0713	0.0717	0.0664	0.0687	0.0699	0.0653	0.0642	0.0668	0.0655	0.0539	0.0590	0.0661
	*N* = 120(*n* = 3, *L* = 40)	0.1	0.0021	0.0022	0.0024	0.0024	0.0025	0.0024	0.0021	0.0022	0.0025	0.0012	0.0016	0.0024
		0.6	0.0493	0.0531	0.0581	0.0569	0.0574	0.0584	0.0511	0.0534	0.0578	0.0305	0.0395	0.0581
	*N* = 120(*n* = 5, *L* = 24)	0.1	0.0023	0.0023	0.0024	0.0022	0.0023	0.0024	0.0018	0.0020	0.0025	0.0009	0.0013	0.0024
		0.6	0.0565	0.0569	0.0581	0.0546	0.0559	0.0584	0.0474	0.0502	0.0578	0.0274	0.0352	0.0581
	*N* = 240(*n* = 3, *L* = 80)	0.1	0.0014	0.0015	0.0017	0.0017	0.0016	0.0017	0.0014	0.0015	0.0017	0.0007	0.0011	0.0017
		0.6	0.0469	0.0514	0.0569	0.0555	0.0557	0.0567	0.0491	0.0515	0.0571	0.0260	0.0363	0.0569
	*N* = 240(*n* = 5, *L* = 48)	0.1	0.0016	0.0017	0.0017	0.0015	0.0016	0.0017	0.0012	0.0013	0.0017	0.0006	0.0008	0.0017
		0.6	0.0539	0.0553	0.0569	0.0524	0.0536	0.0567	0.0445	0.0477	0.0571	0.0215	0.0301	0.0569

## Data Availability

In the present study, we used the information about children under 18 years of age with non-hematological disorders such as Beta-Thalassemia and Idiopathic Thrombocytopenic Purpura (ITP) and also children with hematological malignancies including various types of lymphoma and Acute Lymphocytic Leukemia (ALL), registered in Amir Medical Oncology Center during May 2014 to August 2017, as a population of interest.

## Appendix

### A. The Proof of Lemma 1

We have

(A.1) $$\begin{array}{c} 1-H_{\left[d_{j}\right]}\left(t\right)=1-P\left(\min\left(X_{\left[d_{j}\right]i},C_{\left[d_{j}\right]i}\right)\leq t\right)=P\left(\min\left(X_{\left[d_{j}\right]i},C_{\left[d_{j}\right]i}\right)>t\right)=P\left(X_{\left[d_{j}\right]i}>t,C_{\left[d_{j}\right]i}>t\right)=P\left(X_{\left[d_{j}\right]i}>t\right)P\left(C_{\left[d_{j}\right]i}>t\right)=\left[1-F_{\left[d_{j}\right]}\left(t\right)\right]\left[1-G\left(t\right)\right]. \end{array}$$

### B. Algorithm of Simulation Scenarios

The steps of simulation study algorithm are as follows:

*Step 1: Perform data generation in the following ways:*

- *Generate 1000 random event time observations from the desired distribution (X)*
- *Generate 1000 random censored time observations from the desired distribution (C)*
- *Observe the status variables (δ* = *I*(*X* < *C*))
- *Calculate survival time variable (T* = min (*X*, *C*))

*Step 2: Perform sampling in the different studied designs:*

- *Generate PROS, RSS, and SRS samples from the target population. For PROS and RSS, we generate the samples based on different values for subsetting error matrices, set sizes, and cycle sizes*

*Step 3: Estimate the desired estimators:*

- *estimate the KM estimator using the corresponding formula coding*

*Step 4: calculate comparison criteria:*

- *Compute the MSE of the KM estimator in different percentile points*
- *Compute the MISE values for KM estimators under the three different sampling designs*

*Step 5: Repeat all the above steps 5000 times.*

*Step 6: Compute the mean of 5000 calculated MSE and MISE and report them.*

## Supplementary Materials

Supplementary Materials This supplementary file includes the data for the Section 6 (real data) example in the paper. This file contains the information of children under 18 years of age with nonhematological disorders such as Beta-Thalassemia and Idiopathic Thrombocytopenic Purpura (ITP) and children with hematological malignancies including various types of lymphoma and Acute Lymphocytic Leukemia (ALL), registered in Amir Medical Oncology Center during May 2014 to August 2017. The dataset contains the survival information of 61 patients.
